# HOXA9 gene inhibits proliferation and differentiation and promotes apoptosis of bovine preadipocytes

**DOI:** 10.1186/s12864-024-10231-3

**Published:** 2024-04-11

**Authors:** Lixia He, Xue Feng, Chunli Hu, Shuang Liu, Hui Sheng, Bei Cai, Yun Ma

**Affiliations:** College of Animal Science and Technology, Key Laboratory of Ruminant Molecular and Cellular Breeding of Ningxia Hui Autonomous Region, Ningxia University, 750021 Yinchuan, China

**Keywords:** HOXA9, Bovine, Fat deposition, Differentiation, Proliferation

## Abstract

**Background:**

Hox gene family is an important transcription factor that regulates cell process, and plays a role in the process of adipocytes differentiation and fat deposition. Previous transcriptome sequencing studies have indicated that the Homeobox A9 gene (*HOXA*9) is a candidate gene for regulating the process of bovine lipid metabolism, but the function and specific mechanism of action remain unclear. Therefore, this study aims to explore the role of *HOXA*9 in the proliferation, differentiation and apoptosis of bovine preadipocytes through gain-of-function and lose-of-function.

**Result:**

It found *HOXA*9 highly expressed in bovine adipose tissue, and its expression level changed significantly during adipocytes differentiation process. It gave a hint that *HOXA*9 may be involved in the process of bovine lipid metabolism. The results of *HOXA*9 gain-of-function experiments indicated that *HOXA*9 appeared to act as a negative regulator not only in the differentiation but also in the proliferation of bovine preadipocytes, which is mainly reflected that overexpression of *HOXA*9 down-regulate the mRNA and protein expression level of PPARγ, CEBPα and FABP4 (*P* < 0.05). The mRNA expression level of *CDK*1, *CDK*2, *PCNA*, *CCNA*2, *CCNB*1, *CCND*1 and *CCNE*2, as well as the protein expression of CDK2 also significantly decreased. The decrease of lipid droplets content was the main characteristic of the phenotype (*P* < 0.01), which further supported the evidence that *HOXA*9 was a negative regulator of preadipocytes differentiation. The decrease of cell proliferation rate and EdU positive rate, as well as the limitation of transition of preadipocytes from G0/G1 phase to S phase also provided evidence for the inhibition of proliferation. Apart from this above, we noted an interesting phenomenon that overexpression of *HOXA*9 showed in a significant upregulation of both mRNA and protein level of apoptosis markers, accompanied by a significant increase in cell apoptosis rate. These data led us not to refute the fact that *HOXA*9 played an active regulatory role in apoptosis. *HOXA*9 loss-of-function experiments, however, yielded the opposite results. Considering that *HOXA*9 acts as a transcription factor, we predicted its target genes. Dual luciferase reporter assay system indicated that overexpression of *HOXA*9 inhibits activity of *PCNA* promoter.

**Conclusion:**

Taken together, we demonstrated for the first time that *HOXA*9 played a role as a negative regulatory factor in the differentiation and proliferation of preadipocytes, but played a positive regulatory role in apoptosis, and it may play a regulatory role by targeting *PCNA.* This study provides basic data for further exploring the regulatory network of intramuscular fat deposition in bovine.

## Introduction

Intramuscular fat (IMF), or marbling, one of the indexes of meat quality, is essential to improve the flavor, juiciness, palatability and color of beef. Exploring the mechanism of IMF deposition is the premise of studying fat deposition [1]. There are two primary mechanisms for the expansion of adipose tissue, one is increasing the number of adipocytes (proliferation), the other is increasing the volume of adipocytes (differentiation) [2]. Fat formation includes two stages. The first stage is the differentiation of embryonic stem cells into mesenchymal stem cells with multiple differentiation potentials and the second stage is the terminal differentiation stage [3]. And these are regulated by the sequential activation of various transcription factors [3]. Peroxisome-proliferator-activated receptor γ (PPARγ), one of the core regulators factors of adipogenesis [4, 5], cooperates with transcription factors such as CCAAT-enhancer binding protein α (C/EBPα) and CCAAT-enhancer binding protein β (C/EBPβ) to induce the expression of Lipoprotein Lipase (LPL), Fatty acid binding protein 4 (FABP4), Perilipin 1 (PLIN1) and other downstream transcription factors, thus to regulate the differentiation of adipocytes [6]. Hox gene family is a highly conserved subgroup of homeobox superfamily, includes 39 genes, which are divided into four gene clusters: Hoxa, Hoxb, Hoxc and Hoxd. There are highly conserved gene sequences and similar gene functions among different species and genera [7]. Hox gene family not only participates in the browning of white adipose tissue and the thermogenesis of brown adipose tissue [8, 9], but also regulates the development of adipose tissue in different parts of the body [10, 11], which is an important transcription factor family for regulating adipose production [12–14]. *HOXA*9, a member of homeobox gene of cluster A, is a basic leucine-zipper transcription factor located on the short arm of the chromosome 7, located at the 5’ end of the Hoxa family and consisting of three exons (IAB, ICD, and II). It was first discovered as a partial transcript in the fetal liver [15], which is well known for its key role in lineage commitment of hematopoietic cells [16]. It not only plays a role in regulatory of cell proliferation, differentiation, apoptosis, tissue and organ formation and individual growth and development [17–19], but also participates in the occurrence and poor prognosis of various malignant tumors in human beings [20–22]. Existing reports show that *HOXA*9 differentially expressed in female abdominal and gluteal subcutaneous adipose tissue, which regulated the deposition of fat in different parts of body [23]. Sadkowski et al. also showed that *HOXA*9 was a key candidate gene for regulating IMF development in pigs and cattle through joint analysis of ATAC-seq and RNA-seq [24, 25], but the specific function and mechanism are not clear. Gain-of-function and lose-of-function are the main way to verify gene function in the field of life science [26]. Overexpression and RNA interference are the most common and direct way to explore gene function [27]. The purpose of this study is to investigate the effects of *HOXA*9 on proliferation, differentiation and apoptosis of bovine preadipocytes through overexpression and RNA interference experiments, and provide basic data for further study on the regulatory network of IMF deposition.

## Materials and methods

### Animal and cell culture

The samples were three 7-day-old Guyuan yellow calves from Guyuan Fumin Agricultural Technology Development Co., Ltd. in Yuanzhou District, Guyuan City, Ningxia Hui Autonomous Region. Animals were killed in a painless way by electric shock and without anesthesia. After slaughter, the tissue samples of heart, liver, spleen, lung, kidney, muscle and back adipose were collected. These tissues were washed with sterile physiological saline, cut into small pieces, put into 1.5 mL centrifuge tubes and stored in liquid nitrogen. At the same time, adipose tissue was stored in phosphate buffer saline (PBS, HyClone, Logan, USA) with 1% penicillin and streptomycin (HyClone, Logan, USA) and brought back to the laboratory. Primary adipocytes were isolated using the tissue-block method. After fascia and blood vessels in the adipose tissue were removed with scissors and tweezers, the adipose tissue was cut into small pieces about 1 mm^3^ and placed in new sterile 90 mm petri dishes. The petri dishes were placed upside down at 37℃, in a 5% CO_2_ incubator 5 h later.

### Vector construction, siRNA chemical synthesis and cell transfection

#### Construction of pcDNA3.1-*HOXA*9 overexpression plasmid and siRNA synthesis

According to the sequence of bovine *HOXA*9 (GenBank: NM_001105617.2) in NCBI database, CDS region of *HOXA*9 was amplified and inserted into pcDNA3.1 vector to construct overexpressed *HOXA*9 plasmid. Meanwhile, three interfering fragments targeting *HOXA*9 were designed and synthesized. Sequences are shown in Table 1. When the cell fusion degree of 6-well plates reached 60-80%, 2.5 µg plasmid DNA or 10 µL siRNA were transfected into each well. All transfection experiments were carried out according to the Lipofectamine™ 3000 Reagent USER GUIDE (Thermo Scientific, California, USA), but the cell culture medium was not changed after transfection.

**Table 1:** siRNA sequence information

Sequence number	Name	Sequence	
		Sense (5’-3’)	Antisense (5’-3’)
1	bta*HOXA*9-102	GGCAACUACUACGUGGACUTT	AGUCCACGUAGUAGUUGCCTT
2	bta*HOXA*9-558	GGUUCUCCUCCAGUUGAUATT	UAUCAACUGGAGGAGAACCTT
3	bta*HOXA*9-727	ACCAAACGCUGGAACUAGATT	UCUAGUUCCAGCGUUUGGUTT
4	Control	UUCUCCGAACGUGUCACGUTT	ACGUGACACGUUCGGAGAATT

#### Construction of dual-luciferase reporter plasmids and dual luciferase assay

The partial promoter of *PPARγ* (GenBank: NC_037349.1), *CDK*2 (GenBank: NC_037332.1), *FABP*4 (GenBank: NC_037341.1), and *PCNA* (GenBank: NC_037340.1) genes were cloned into pGL3-basic plasmid vector to construct dual luciferase reporter plasmids. When the cell fusion degree of 24-well plates reached 60-80%, 1 µg pcDNA3.1/pcDNA3.1-*HOXA*9, 0.8 µg dual luciferase reporter plasmids DNA, and 20 ng pRL-TK plasmids DNA were transfected into 293T cells. All transfection experiments were carried out according to the Lipofectamine™ 3000 Reagent USER GUIDE, but the cell culture medium was not changed after transfection. All dual luciferase experiments were performed according to the instructions Dual-Luciferase® Reporter Assay System.

### Induced differentiation of preadipocytes and oil red O staining

Then the cell fusion degree of preadipocytes reached 90–100%, the growth medium (GM, DMEM + 10% fetal bovine serum) was replaced with induce medium (IM, GM containing 10 µg/mL of insulin, 1 µmol/L of dexamethasone, 0.5 mmol/L IBMX, and 1 µmol/L of rosiglitazone) to continue culture, after 2 days, IM was replaced with differentiation-maintaining medium (MM, GM containing 10 µg/mL of insulin and 1 µmol/L of rosiglitazone). MM was changed every 2 days and cells were collected after 8 days. For Oil Red O staining, mature adipocytes were washed with PBS and fixed with 4% formaldehyde solution for 30 min. Subsequently, cells dyed with Oil Red O working solution for 30 min, and decolored with 60% isopropanol. Then, hematoxylin was added to each well to counterstain nucleus, and cells were observed by a fluorescence microscope. Finally, 1 mL of 100% isopropanol was added to each well for quantitative analysis of lipid droplets, and the absorbance value was detected at 490 nm.

### EdU and CCK8 assay

CCK8 kits (Meulunbio, Shanghai, China) and EdU kits (Beeyotime, Shanghai, China) were used to detected to cell proliferation. In CCK8 assay, preadipocytes were placed to 96-well petri dishes. After 0, 24, 48 and 72 h of transfection, 10 µL CCK8 solution was added to each well, and the absorbance at 450 nm was measured with a microplate reader (SYNERGY|LX, BioRad, Hercules, CA, USA) after cells were incubated in the dark for 1 h. For EdU staining, after transfection of plasmids and interfering fragments 48 h, cells were labeled with EdU solution and continued to culture for 6 h. Subsequently, cells were fixed with 4% formaldehyde and infiltrated with 0.3% Triton X-100 for 15 min. Then the click reaction mixture was added to each well and cells were incubated in the dark for 30 min. DAPI was added to dye the nucleus for 10 min, and finally cells were observed and imaged by a fluorescence microscope.

### Flow Cytometry

#### The flow cell cycle

Cell cycle was detected by cell cycle kits (Beyptime, Shanghai, China). Preadipocytes were placed in a 6-well petri dish, after transfection for 48 h, cells were digested with trypsin. Cells were collected in 1.5 mL centrifuge tubes and added 1 mL precooled 70% ethanol. Kept cells stored refrigerator at 4 °C for 24 h. Then, the ethanol was removed and 1 mL PBS was added to wash cells. Subsequently, 0.5 mL propidium iodide staining solution was added to each tube of cells. Cells were resuspended and incubated in the dark at 37 °C for 30 min. Finally, cells were detected by flow cytometry (BD-C6 Plus, 2000).

#### Flow apoptosis

Apoptosis was detected by Annexin V-FITC cell apoptosis detection kits (Beyptime, Shanghai, China). Growth medium were collected into centrifuge tubes. Cells were digested with trypsin, 3 min later, the previously collected growth medium was added to the 6-well plates to stop digesting and cells were collected to centrifuge tubes. After centrifugation at 1000 rpm for 5 min, cells were resuspended with PBS buffer and stimulated in 50℃ water for 2–3 min. Centrifuge again, discarded the supernatant and resuspend cells. Then, 195 µL Annexin V-FITC was added into a tube, cells were resuspended gently and added 5 µL Annexin V-FITC. Finally, 10 µL propidium iodide staining solution was added into a tube. The apoptotic cells were detected by a flow cytometry (each treatment has three parallel replicates) after incubated 20 min in the dark at room temperature.

### RNA extraction, cDNA synthesis and RT-qPCR

Total RNA was extracted from tissues and cells by TRIzol method. The operation was carried out according to the instructions of TRIzol kits (Vazyme, Nanjing, China). The concentration (ng/µL) and the value of OD_260/280_ of RNA were detected by a multifunctional full-wavelength enzyme-labeled instrument (SYNERGY|LX), and the quality of RNA was detected by 1% agarose gel electrophoresis. 100 ng RNA was reversely transcribed into cDNA according to the instructions of reverse transcription kits (TaKaRa, Kyoto, Japan). cDNA was used as a template (three parallel replicates were set for each biological sample), *GAPDH* as a standardized reference gene. RT-qPCR was performed according to the instructions of ChamQ Universal SYBR qPCR Master Mix (Vazyme, Nanjing, China) (three parallel replicates for each treatment). Primer information is shown in Table 2.

**Table 2:** Primer information

Gene	Primer sequences (5’-3’)	Products size/bp	Annealing temperature/℃
*HOXA*9	F:CCACGCTTGACACTCACACTT R:GCCGCTCTCATTCTCAGGATTG	122	60
*GAPDH*	F:CCAACGTGTCTGTTGTGGAT R:CTGCTTCACCACCTTCTTGA	80	60
*PPARγ*	F:AGGATGGGGTCCTCATATCC R:GTCAGCTCTTGGGAACGGAA	137	60
*CEBPα*	F:TGGACAAGAACAGCAACGAG R:TTGTCACTGGTCAGCTCCAG	130	60
*CEBPβ*	F:TTCCTCTCCGACCTCTTCTC R:CCAGACTCACGTAGCCGTACT	79	60
*FABP*4	F: AAGTCAAGAGCATCGTAA R:CCAGCACCATCTTATCAT	111	60
*LPL*	F:ACGATTATTGCTCAGCATGG R:ACTTTGTACAGGCACAACCG	130	60
*CDK*1	F:GAAGGGGTTCCTAGTACTGC R:ATGAACTGACCAGGAGGG	176	60
*CDK*2	F:ATGAACTGACCAGGAGGG R:GCCAGGAGTTACTTCTATGC	115	60
*PCNA*	F:GAACCTCACCAGCATGTCCA R:TACTAGTGCCAACGTGTCCG	97	60
*CCNA*2	F:ACACAGTCACAGGACAAAGC R:TCTGAGGTAGGTCTGGTGAA	107	60
*CCNB*1	F:TGGAGAGGTTGATGTTGAGC R:TCTGAGAAGGAGGAAAGTGC	95	60
*CCND*1	F:TGGTCCTGGTGAACAAACTC R:ATCTGCTTGTTCTCCTCGGC	106	60
*CCNE*2	F:GCTTATGTCACTGATGGTGCTTG R:TTAGCCAGGAGATGACCGTTAC	112	60
*BAX*	F:GAGATGAATTGGACAGTAACA R:TTGAAGTTGCCGTCAGAA	118	60
*BAD*	F:TCCCAGAGTTTGAGCAGAGTGA R:TTAGCCAGTGCTTGCTGAGAC	108	60
*BCL*2	F:ATGTGTGTGGAGAGCGTCAA R:GAGACAGCCAGGAGAAATCA	181	60

### Western blot

Cells protein were extracted by whole protein extraction kits (Epyzime, Shanghai, China). Cells were washed with PBS buffer, and 150 µL protein lysis (1% PMSF) was added to each well, which was collected in a 1.5 mL centrifuge tube after 5 min. Subsequently, cells were shaken with vortex for 30 s, kept them on ice 5 min. This operation was repeated 5 times. Later, cells were centrifuged at 12,000 r for 5 min. Then supernatant was placed in new centrifuge tubes. The BCA Protein Assay Kits (Epyzime, Shanghai, China) were used to determine protein concentration. 5× protein loading buffer (Epyzime, Shanghai, China) was added to the protein samples, boiled 10 min and stored them at -80℃. After prepared concentrated gel and separated gel (PAGE Gel Fast Preparation Kits, Epyzime, Shanghai, China), the protein samples subjected to electrophoresis, transfered to polyvinylidene fluoride membrane, sealed, primary antibody incubation and secondary antibody incubated. Finally, they were visualized by enhanced chemiluminescence (ECL) detection systems (Epyzime, Shanghai, China). The brands and dilution ratio of primary antibody and secondary antibody are showed in Table 3.

**Table 3:** Information of primary antibody and secondary antibody

Name	Brand	Size/kDa	Dilution rate
FABP4 Antibody	Abways (CY6768)	15	l:500
GAPDH Antibody	Abways (AB0036)	36	l:3000
PPARα Rabbit Polyclonal Antibody	Beyotime (AF7794)	57	l:500
CDK2 Antibody	Abways (CY5020)	33	l:500
Anti-CEBPA (Phospho-Ser21) rabbit polyclonal antibody	Sangon Biotec (D151230)	38	1:500
HOXA9 Rabbit pAb	Abclonal (A1908)	35	1:500
Bax Antibody	Abways (CY5059)	21	l:1000
Goat anti-Rabbit IgG	Sangon Biotec (D111018)		1:20000

### Data analysis

One-way analysis of variance (one-way ANOVA) using GraphPad Prism 9 software was carried out, and 2^−∆∆*Ct*^ method was used to analyze the results of RT-qPCR. The data were expressed as mean ± standard error (SEM). Among them, * means *P* < 0.05, which means significant difference. ** means *P* < 0.01, which means the difference is extremely significant.

## Results

### Analysis of the expression pattern of *HOXA*9

**Fig. 1:**
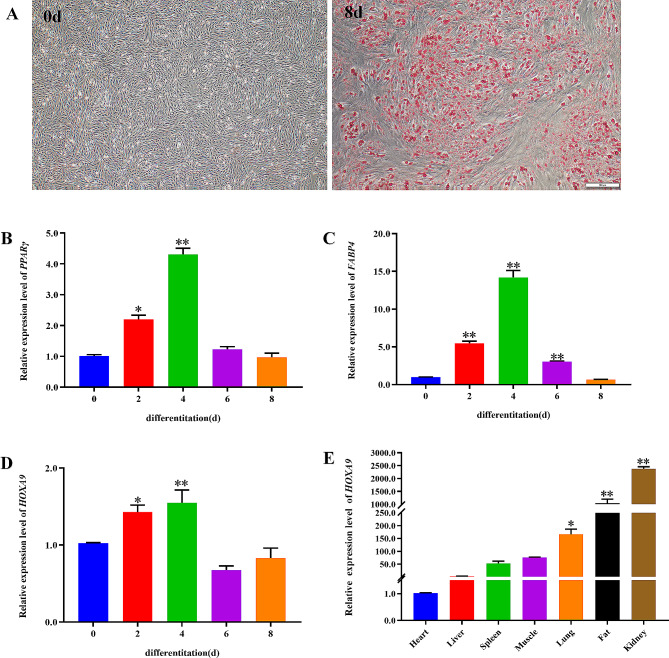
Analysis of the expression pattern of *HOXA*9. (**A**) Oil Red O staining images of preadipocytes induced for 0 d and 8 d. (**B**-**D**) RT-qPCR analysis of the relative level of *PPARγ*, *FABP*4 and *HOXA*9 during preadipocytes differentiation. All date were compared with 0 d. (**E**) RT-qPCR analysis of the relative mRNA level of *HOXA*9 in different tissues of bovine. All data were compared with heart. *GAPDH* as a standardized reference gene. *n* = 3, * *P* < 0.05, ** *P* < 0.01

In order to clarify the role of *HOXA*9 in fat deposition, preadipocytes were isolated and induced to adipogenic differentiation. Oil Red O staining showed that the number and size of the lipid droplets increased significantly during adipocytes differentiation (Fig. 1-A), and the relative expression level of *PPARγ* and *FABP*4 significantly up-regulated (Fig. 1-B, C). Meanwhile, the expression level of *HOXA*9 was changed significantly during adipocytes differentiation (Fig. 1-D) and highly expressed in fat (Fig. 1-E). All the above date indicated that the adipocytes differentiation system was successfully established and *HOXA*9 may regulate the adipogenic differentiation of bovine preadipocytes.

### *HOXA*9 inhibits the differentiation of bovine adipocytes

**Fig. 2:**
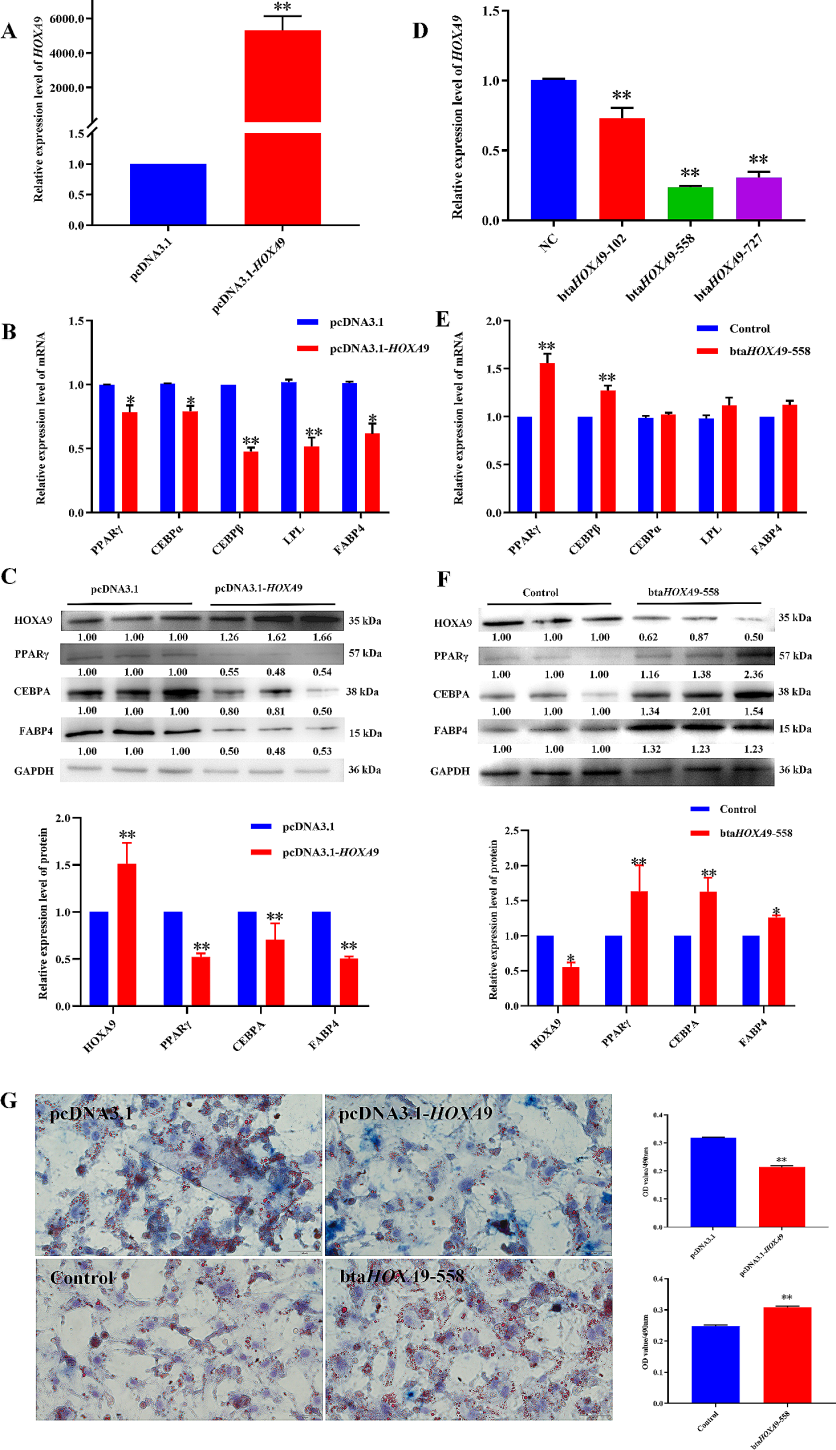
*HOXA*9 inhibits the differentiation of bovine adipocytes.(**A**.**D**) Relative expression level of mRNA after overexpression/interference of *HOXA*9. (**B**.**E**) Relative expression level of mRNA of adipogenic markers after overexpression/interference of *HOXA*9. (**C**.**F**) Relative expression level of protein of adipogenic markers after overexpression/interference of *HOXA*9. (**G**) Oil Red O staining images (scale bar = 50 μm) of preadipocytes after overexpression/interference of *HOXA*9. *GAPDH* as a standardized reference gene. The data were expressed as mean ± SEM, *n* = 3, * *P* < 0.05, ** *P* < 0.01

In order to explore the role of *HOXA*9 in adipocytes differentiation, pcDNA3.1-*HOXA*9 and pcDNA3.1 plasmids were transfected into preadipocytes to overexpress *HOXA*9. Results showed that the overexpression efficiency was more than 4000 times (Fig. 2-A, *P* < 0.01), achieving the high expression of *HOXA*9 in adipocytes. The high expression of *HOXA*9 in adipocytes not only decreased the mRNA expression level of *PPARγ*, *CEBPα*, *CEBPβ*, *LPL* and *FABP*4 (Fig. 2-B, *P* < 0.05), but also significantly decreased the protein expression level of FABP4, CEBPα and PPARγ (Fig. 2-C). Meanwhile, Oil Red O staining showed that the number and concentration of lipid droplets decreased significantly (Fig. 2-G, P *<* 0.01), of which were caused by overexpression of *HOXA*9. Furthermore, the interference experiments were carried out on the *HOXA*9. Three interference sequences (bta*HOXA9*-102, bta*HOXA*9-558 and bta*HOXA*9-727) targeting the *HOXA*9 were designed and transfected into preadipocytes to silence *HOXA*9 expression. RT-qPCR was used to measure the knockdown efficiency. It found that, compared to NC group, bta*HOXA*9-558 has the strongest knockdown efficiency more than 60% (Fig. 2-D, *P* < 0.01), so bta*HOXA*9-558 was selected for subsequent experiments. It showed that the mRNA expression level of adipogenic markers of *PPARγ* and *CEBPβ* (Fig. 2-E, *P* < 0.01) and the protein expression level of FABP4, CEBPα and PPARγ were significantly increased (Fig. 2-F) after transfection of bta*HOXA*9-558 and induced differentiation. Coincidently, Oil Red O staining also showed that the number and aggregation lipid droplets increased significantly (Fig. 2-G, *P* < 0.01).

### *HOXA*9 inhibits the proliferation of bovine adipocytes

**Fig. 3:**
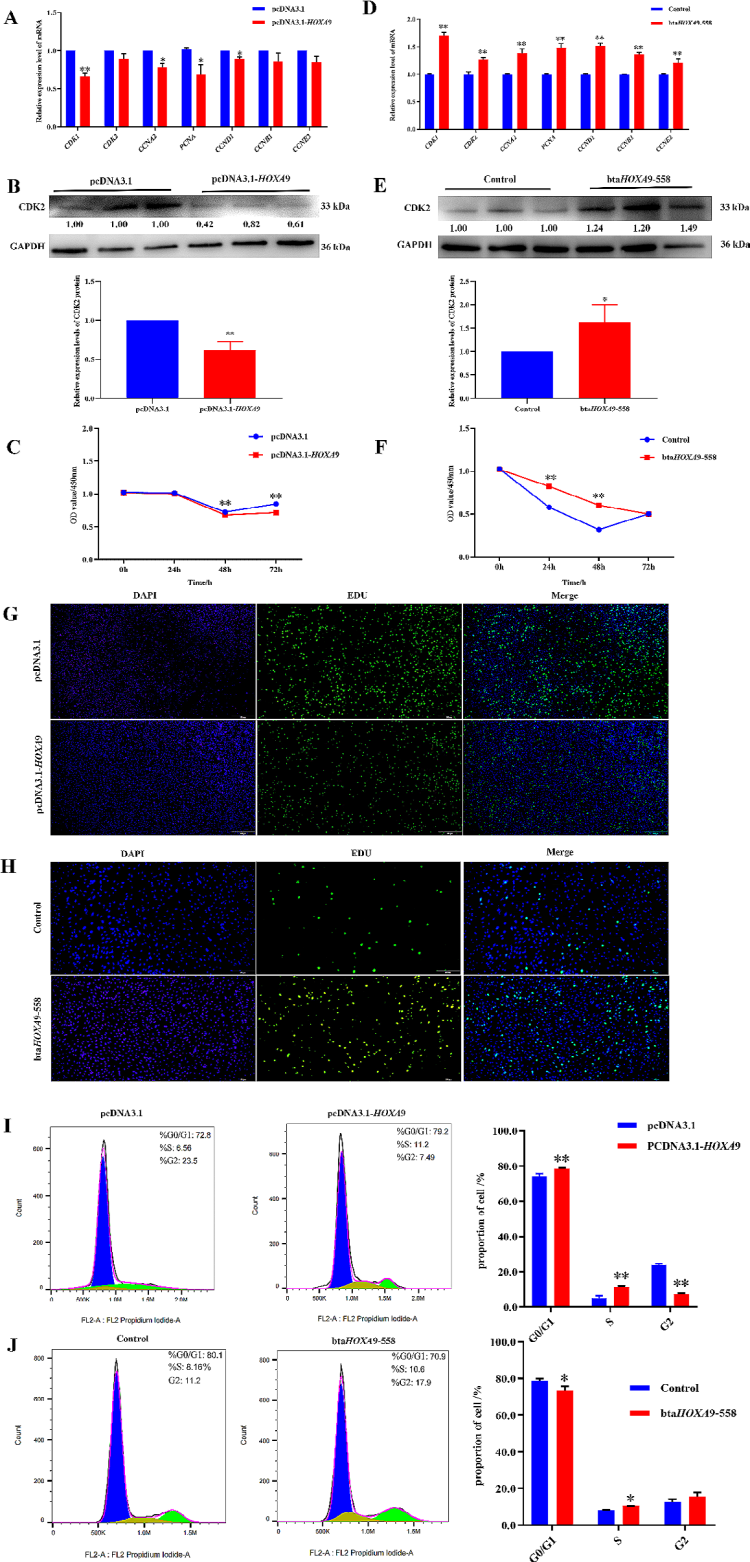
*HOXA*9 inhibits the proliferation of bovine adipocytes. (**A**.**D**) Relative expression level of mRNA of proliferation markers after overexpression/interference of *HOXA*9. (**B**.**E**) Relative expression level of protein of proliferation markers after overexpression/interference of *HOXA*9. (**C**.**F**) The viability of cells was detected by CCK8 after overexpression/interference of *HOXA*9. (**G**.**H**) Cell proliferation rate was detected by EdU (scale bar = 500 μm) after overexpression/interference of *HOXA*9. (**I**.**J**) The cell cycle analysis after overexpression/interference of *HOXA*9. *GAPDH* as a standardized reference gene. The data were expressed as mean ± SEM, *n* = 3, * *P* < 0.05, ** *P* < 0.01

We have studied the role of *HOXA*9 in differentiation, so next to further validated the role of *HOXA*9 in regulating proliferation of bovine adipocytes. First of all, RT-qPCR showed that the relative mRNA expression level of the proliferation-related genes *CDK*1, *CCNA*2, *PCNA* and *CCND*1 were significantly decreased (Fig. 3-A, *P* < 0.05), and the protein expression level of CDK2 was also significantly decreased with transfection of pcDNA3.1-*HOXA*9 (Fig. 3-B, *P* < 0.05). Then, Cell proliferation was detected by CCK8 and EdU staining. The results showed that overexpression of *HOXA*9 significantly reduced the proliferation rate and EdU positive rate of preadipocytes (Fig. 3-C, G). Subsequently, we found that the cell cycle arrested in G0/G1 and S phases after overexpression of *HOXA*9, and the proportion of cells in G2 phase decreased significantly (Fig. 3-I, *P* < 0.05) through flow cytometry analysis. Meanwhile, the interference experiments showed opposite effect to the overexpression experiments. The relative expression level of mRNA and protein of proliferation markers were significantly increased (Fig. 3-D, E. *P* < 0.01), the cell viability was significantly higher than that of the control group at 24 and 48 h (Fig. 3-F. *P* < 0.01), and the positive rate of EdU was also significantly increased (Fig. 3-H) with transfection of bta*HOXA*9-558. Furthermore, results of flow cycle showed that the number of cells in G0/G1 phase decreased, and the proportion of cells in S phase and G2 phase increased after interference, which promoted cell proliferation (Fig. 3-J, *P* < 0.05).

### *HOXA*9 promotes the apoptosis of bovine preadipocytes

**Fig. 4:**
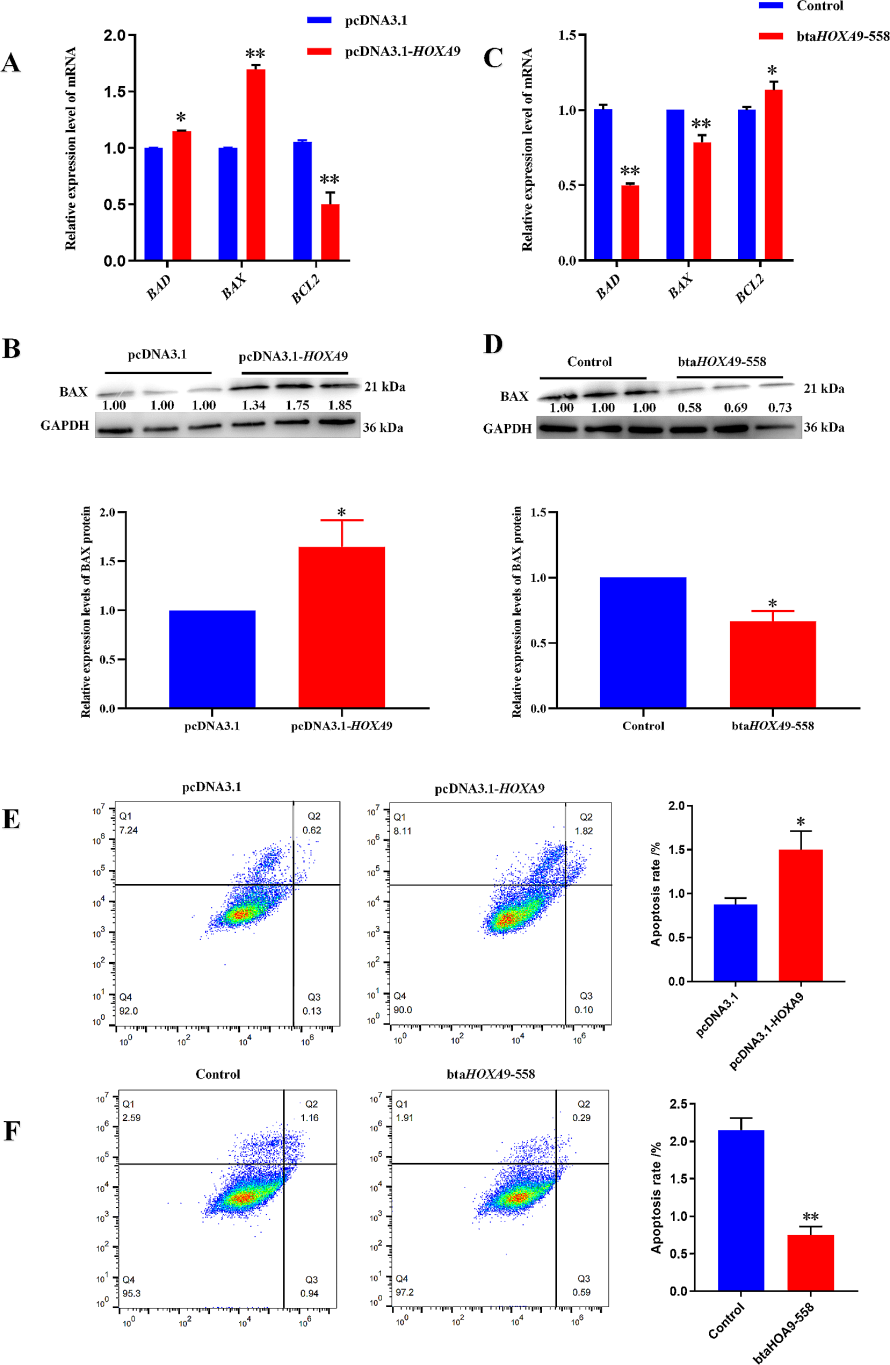
*HOXA*9 promotes the apoptosis of bovine preadipocytes. (**A**.**C**) Relative expression level of mRNA of apoptosis-related genes after overexpression/interference of *HOXA*9. (**B**.**D**) Relative expression level of protein of apoptosis-related genes after overexpression/interference of *HOXA*9. (**E**.**F**) The apoptosis was detected by flow cytometry after overexpression/interference of *HOXA*9. *GAPDH* as a standardized reference gene. The data were expressed as mean ± SEM, *n* = 3, * *P* < 0.05, ** *P <* 0.01

To further explore the role of *HOXA*9 on adipocytes apoptosis. RT-qPCR and WB results showed overexpression of *HOXA*9 up-regulate the relative mRNA expression level of apoptosis markers *BAD* and *BAX* and the expression level of BAX protein, and down-regulate the relative mRNA expression level of anti-apoptosis gene *BCL*2 (Fig. 4-A, B). But results were reversed with silence *HOXA*9 (Fig. 4-C, D). Meanwhile, Flow cytometry analysis showed that transfection of pcDNA3.1-*HOXA*9 plasmid significantly increased the apoptosis rate (Fig. 4-E), while the number of apoptosis cells decreased after interference (Fig. 4-F).

### *HOXA*9 may target the *PCNA* promoter

**Fig. 5:**
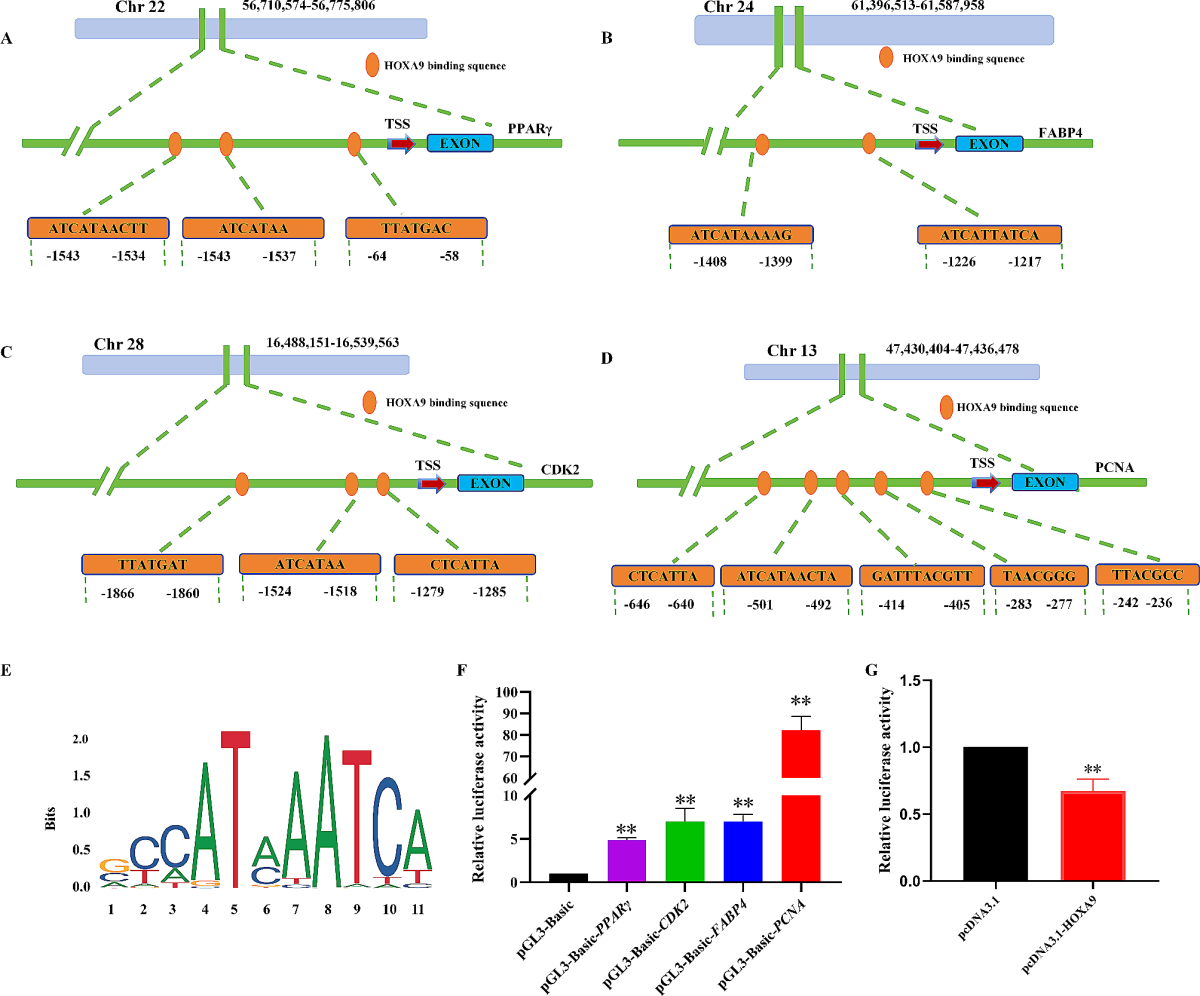
*HOXA*9 may target the *PCNA* promoter. (**A**-**D**) Prediction of *HOXA*9 binding sites. (**E**) *HOXA*9 transcription-binding motif. (**F**) The promoter activity of marker genes were detected by dual luciferase reporter assay system. (**G**) Overexpression of *HOXA*9 affected the activity of *PCNA* promoter

*HOXA*9 inhibited the proliferation and differentiation of preadipocytes and promoted apoptosis by up-regulating and down-regulating multiple marker genes, we hypothesized that those may be potential targets of *HOXA*9. To verify this conjecture, the *HOXA*9 transcriptional binding motif (Fig. 5-E) and sites was first analyzed by bioinformatics (Fig. 5-A, B, C, D). Following pGL3-basic-*PPARγ*, pGL3-basic-*CDK*2, pGL3-basic-*FABP*4 and pGL3-basic-*PCNA* promoter dual luciferase reporter plasmids were transfected into 293T Cells, *PCNA* promoter had the most relative luciferase activity (Fig. 5-F). Further cotransfection of pcDNA3.1, pcDNA3.1-*HOXA*9 and pGL3-basic-*PCNA* showed that overexpression of *HOXA*9 significantly reduced relative luciferase activity (Fig. 5-G). It was indicated that *HOXA*9 may target the *PCNA* promoter to regulate adipocytes proliferation.

## Discussion

Fat deposition is regulated by a complex signal transduction network formed by transcription factors, enzymes, hormones and signal pathways. Transcription factors control the transcription process by combining specific DNA sequences and play an important regulatory role in the development and deposition of fat [28]. *HOXA*9 is an important transcription factor, which is involved in the regulation of organ and morphogenesis, differentiation and adhesion, migration and cell cycle [29–31]. Previous studies showed that *HOXA*9 expression is significantly up-regulated in subcutaneous adipose tissues after bariatric surgery [34], which played an important role in regulating the function of adipose tissue. Sadkowski and others have reported that *HOXA*9 was a candidate gene for regulating intramuscular fat deposition of bovine [25]. In this study, *HOXA*9 was found to be abundant in adipose tissue, and the expression level of *HOXA*9 changed significantly during preadipocytes differentiation. It suggested that *HOXA*9 may play a key regulatory role in adipogenesis of bovine. Therefore, this study aimed to investigate the function of *HOXA*9 gene in ruminantia fat deposition.

As we all know, the differentiation of adipocytes involves cascaded regulation of multiple transcription factors. *PPARγ* is the core of regulation of adipocytes differentiation [32]. Ectopic expression of *CEBPβ* and *CEBPδ* bound to the *PPARγ* promoter to induce expression of PPARγ/RXR [33, 34], which in turn activated *CEBPα*, both of them form a positive feedback that initiated/maintained the differentiation of adipocytes and activated downstream genes [35, 36]. *LPL* and *FABP*4, as downstream genes of *PPARγ*, directly or indirectly affected by *PPARγ* to maintain lipogenesis [37, 38]. These genes were considered as markers of adipocytes differentiation. In this study, we found that *HOXA*9 was a negative transcription factor for fat deposition, which down-regulated not only the mRNA of adipogenic markers of *PPARγ*, *CEBPα*, *CEBPβ, FABP4* and *LPL*, but also the protein expression of PPARγ, CEBPα and FABP4 at the molecular level. At the same time, overexpression of *HOXA*9 inhibited the number and aggregation of lipid droplets that inhibited adipocytes differentiation at the morphological level. Until now, there is no research about function and specific mechanism of *HOXA*9 for adipocytes differentiation in the existing literature, but some studies found that silence of *HOXA*9 promoted the differentiation of leukemia cells [18] and inhibited the differentiation of normal myeloid progenitor by inhibiting the activity of *CEBPα* gene + 8 kb enhancer [39]. Whether it has function in adipocytes remain to be explored.

The basis of lipogenesis is the increase of cell number and the accumulation of lipid droplets, so we further explored the effect of *HOXA*9 on proliferation and differentiation of preadipocytes. We revealed that *HOXA*9 negatively regulated the proliferation of adipocytes. *PCNA*, an auxiliary factor for the replication polymerases δ and ε (Pol δ and Pol ε), was a key factor in DNA replication and cell cycle regulation [40], existing mainly as a homotrimer whose expression increased in the late G1 to S phases of the cell cycle immediately before DNA synthesis [41]. *HOXA*9 bound to p21, cyclin D, and Gadd45 to regulate cell cycle progression [42, 43]. And it also bound to human DNA-(cytosine-5) methylchainase (MCMT) [44]. In this study, dual luciferase reporter assay system showed that overexpression of *HOXA*9 affected promoter activity of *PCNA.* We speculated that *HOXA9* may target the *PCNA* promoter to regulate its expression and inhibit adipocytes proliferation. Meanwhile, *HOXA*9 was also an inhibitor of vascular smooth muscle cells (VSMC) proliferation and migration. After silencing, it reduced the expression of synthetic proteins (osteocalcin and PCNA) and enhanced the expression of contractile proteins (α-SMA and SM22α). Thus, it inhibited the proliferation of muscle cells mediated by ox-LDL [45]. However, *HOXA*9 regarded as the “switch” of cell proliferation in the process of myeloid leukemia, which can promote the expression of *CDK*6, *CyclinD*1 gene and telomerase RNA by triggering pleiotropic oncogenes *Myc* and *Myb*, and provide necessary cofactors to maintain the rapid proliferation of cells [46]. Moreover, the protein complex formed by *HOXA*9 and *C/EBPα* targeted *Cdkn2a/b* to overcome G1 phase blockage, promoted the proliferation of bone marrow cells and advanced the process of myeloid leukemia [47]. *HOXA*9 acted as a cancer promoter in head and neck squamous cell carcinoma (HNSCC) and laryngeal squamous cell carcinoma to promote the proliferation and migration of cancer cells [48, 49]. Therefore, *HOXA*9 played different regulation functions for proliferation between normal somatic cells and cancer cells. At the same time, this study also revealed that *HOXA*9 gene was a positive regulator of adipocytes apoptosis. Overexpression of *HOXA*9 significantly promoted the mRNA expression of *BAD* and *BAX* genes, reduced the expression of *BCL*2 gene, and increased the apoptosis rate. Moreover, previous studies showed that *HOXA*9 accelerated the apoptosis process of primary muscle satellite cells by affecting atrophic Signaling pathways [50] and negatively regulated downstream anti-apoptosis and autophagy-promoting genes (including *BCL-XL*, *ULK*1, *ATG*3 and *ATG*12) of NF-κB to promote the apoptosis of skin squamous cell carcinoma (cSCC) cells and inhibit autophagy [51]. This meant *HOXA*9 promoted cells apoptosis. Our results were consistent with the findings of these studies. In this study, we found that *HOXA*9 not only inhibited the accumulation of lipid droplets in phenotype, but also inhibited adipocytes proliferation at the molecular level, possibly by targeting *PCNA*. Meanwhile it promoted the process of adipocytes apoptosis, which was a negative regulator of fat deposition. However, the specific mechanisms remain to be further explored, especially the signaling pathway.

## Conclusion

**Fig. 6:**
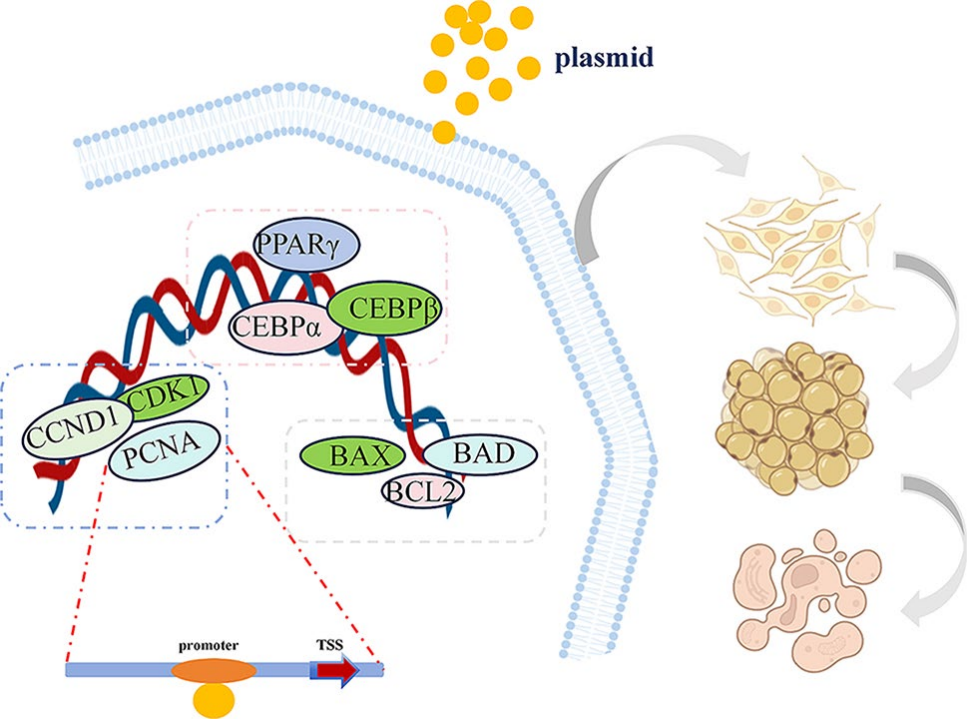
The mechanism of *HOXA*9 regulating fat deposition

In a word, in this study, we confirmed that the *HOXA*9 has the ability to inhibit the proliferation and differentiation of adipocytes and promote apoptosis. It may play a regulatory role by targeting *PCNA* and it is a negative regulator of fat deposition for the first time. Therefore, *HOXA*9 gene may become a new key factor to regulate bovine fat deposition (the mechanism is shown in Fig. 6). This study expanded the key genes bank to explore the regulatory network of bovine fat deposition.

## Data Availability

The data presented in this study are available in the article.
